# The immune-related plasma protein LAT2 as a protective modulator in diabetic retinopathy: a Mendelian randomization study

**DOI:** 10.3389/fendo.2025.1638661

**Published:** 2025-07-17

**Authors:** Ming Yang, Weizhen Wu

**Affiliations:** Department of Ophthalmology, Beijing Friendship Hospital, Capital Medical University, Beijing, China

**Keywords:** diabetic retinopathy, Mendelian randomization (MR), biomarker, LAT2, phenome-wide MR

## Abstract

**Background:**

Diabetic retinopathy (DR) is a leading cause of vision loss worldwide. Although numerous observational studies have explored candidate biomarkers, the causal contributions of circulating plasma proteins to DR pathogenesis remain largely unclear due to confounding and reverse causality.

**Methods:**

To address this, we performed a two-sample Mendelian randomization (MR) analysis using protein quantitative trait loci (pQTLs) derived from the UK Biobank Pharma Proteomics Project (n = 54,219) and DR outcome data from the FinnGen cohort (n = 96,429; 14,142 cases). Colocalization and transcriptome-based MR analyses were conducted to validate causal protein candidates. We further performed experimental validation in hyperglycemia-induced retinal cells and assessed immune mediation using MR-based mediation analysis. A phenome-wide MR (MR-PheWAS) was also conducted to evaluate disease specificity.

**Results:**

Among five significant proteins, we identified Linker for Activation of T Cells Family Member 2 (LAT2) as a robust protective factor for DR (OR = 0.358, 95% CI: 0.215–0.597, p < 0.001). Colocalization analysis (PP.H4 = 0.8546) and SMR analysis supported a shared genetic basis between LAT2 expression and DR. LAT2 expression was significantly upregulated under high-glucose stress in retinal cells. Mediation MR revealed that CD27^+^ switched memory B cells partially mediated the protective effect of LAT2 (mediation proportion: 6.2%, *p* = 0.047). The MR-PheWAS further confirmed the tissue-specific association of LAT2 with DR.

**Conclusions:**

LAT2 may be a potential protective factor for diabetic retinopathy, offering preliminary insight for future biomarker development and prevention strategies.

## 1 Introduction

Diabetic retinopathy (DR) is a common and sight-threatening microvascular complication of diabetes mellitus, representing a leading cause of visual impairment and blindness among working-age adults worldwide (1–3). Its pathogenesis involves a complex interplay of hyperglycemia-induced oxidative stress, inflammation, and vascular dysfunction, ultimately leading to retinal ischemia and neovascularization (4, 5). Despite advances in clinical management, including glycemic control and intravitreal therapies, the disease burden of DR remains substantial. Current treatments predominantly target advanced stages, while effective strategies for early prediction and prevention remain limited.

Given the multifactorial nature of DR, identifying upstream molecular drivers and reliable biomarkers is of critical importance. Circulating proteins, especially those involved in immune regulation and vascular homeostasis, have emerged as promising candidates (6, 7). However, traditional observational studies are often limited by residual confounding and reverse causality, hindering the establishment of causal relationships between biomarkers and disease risk.

**Figure 1:**
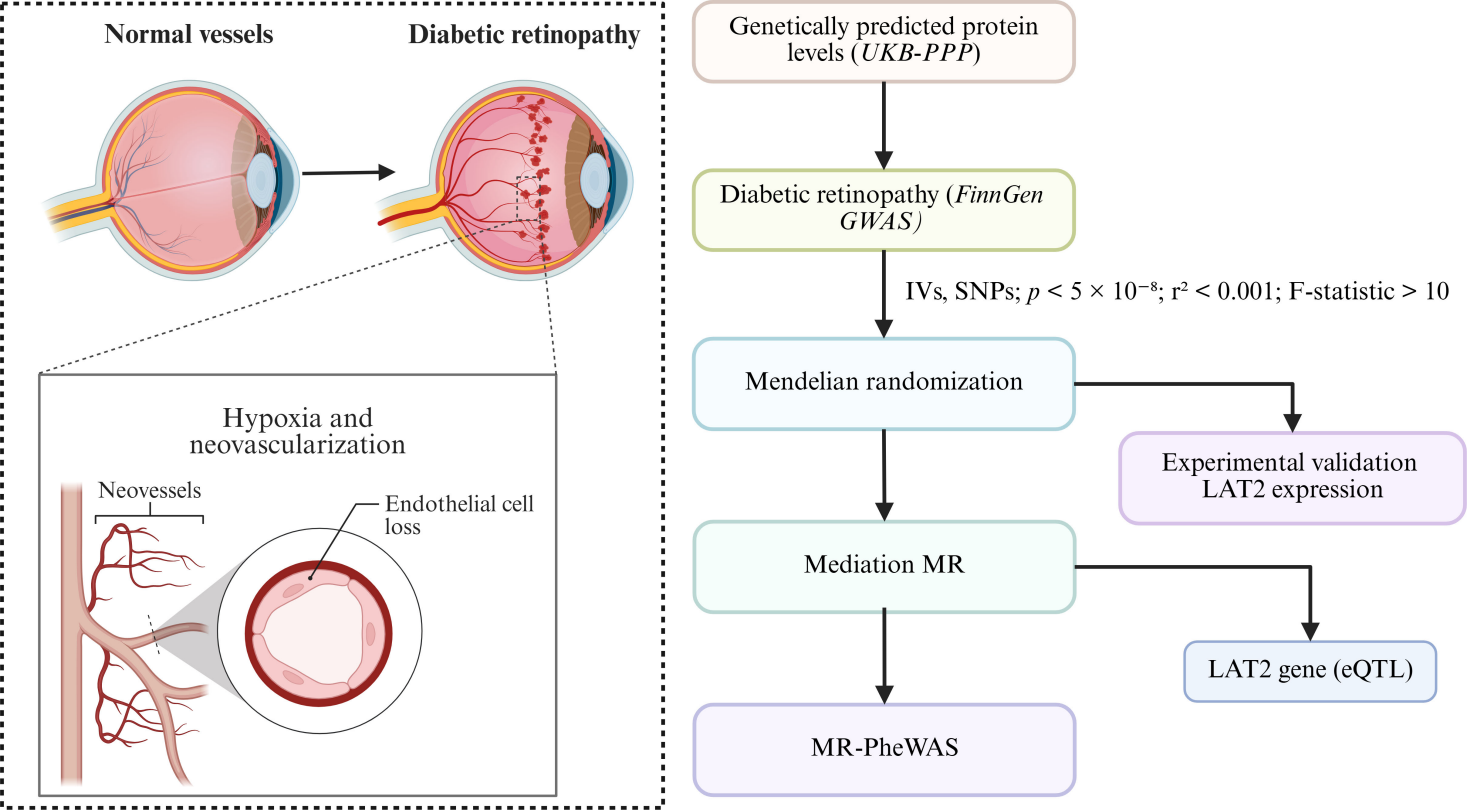
Overview of study rationale and analysis workflow. This figure presents an overview of the study design. The left panel depicts the pathological changes in diabetic retinopathy (DR), including neovascularization and endothelial cell loss resulting from chronic hyperglycemia-induced hypoxia. The right panel illustrates the analytical workflow, starting from genetically predicted plasma protein levels (derived from the UK Biobank Pharma Proteomics Project, UKB-PPP) and DR outcome data (from FinnGen GWAS). Mendelian randomization (MR) was performed using instrumental variables (IVs) selected under stringent criteria (p < 5 × 10^−8^, r² < 0.001, F-statistic > 10). Subsequent analyses included colocalization and expression quantitative trait loci (eQTL) analysis to validate LAT2 as a causal gene, experimental validation under high-glucose conditions, and mediation MR to assess immune involvement. MR-PheWAS was conducted to explore phenotypic specificity.

To address these limitations, this study employed a two-sample Mendelian randomization (MR) approach to investigate the causal effects of genetically predicted plasma protein levels on the risk of diabetic retinopathy (8–10). MR leverages genetic variants as instrumental variables to infer causality, thereby minimizing the influence of confounders and mitigating reverse causation (11, 12). Furthermore, we integrated colocalization analysis and transcriptome based MR to validate target proteins and explore potential tissue-specific effects. By incorporating mediation MR, we also aimed to elucidate the downstream immunological mechanisms involved in DR pathogenesis. This integrative framework offers novel insights into the molecular etiology of diabetic retinopathy and may contribute to the development of protein-based risk stratification and prevention strategies. The research idea of ​​this study is shown in **Figure 1**.

## 2 Methods

### 2.1 Cell culture and treatment

Human retinal microvascular endothelial cells (hRMECs) and ARPE-19 retinal pigment epithelial cells were obtained from the Cell Bank of the Chinese Academy of Sciences (Shanghai, China). hRMECs were cultured in endothelial cell medium supplemented with 10% fetal bovine serum (FBS) and 1% penicillin-streptomycin. ARPE-19 cells were maintained in DMEM/F12 (Gibco, USA) containing 10% FBS and 100 U/mL penicillin-streptomycin. All cells were incubated at 37°C with 5% CO_2_. To induce hyperglycemia, cells were treated with 30 mM D-glucose for 48 hours (13, 14). Cells cultured in normal glucose (5–5.5 mM) served as controls.

### 2.2 Cell viability assay

Cell viability was assessed using the Cell Counting Kit-8 (CCK-8, Dojindo). hRMECs and ARPE-19 cells were seeded in 96-well plates at 1 × 10^4^ cells/well and incubated overnight. After 48-hour treatment, 10 μL of CCK-8 solution was added per well and incubated at 37°C for 1 hour. Absorbance was measured at 450 nm using a microplate reader.

### 2.3 Quantitative PCR

Total RNA was extracted from ARPE-19 cells using TRIzol reagent (Invitrogen, USA) and reverse-transcribed into cDNA with PrimeScript RT Master Mix (Takara, Japan). The following cycling conditions: 95°C for 30 s, followed by 40 cycles of 95°C for 5 s and 60°C for 30 s. The following primers were used: LAT2 F 5′-GCTGGCTGTGATGTTCTGG-3′, R 5′-TGGTGGTAGAGGTGCTGATG-3′; GAPDH F 5′-GAAGGTGAAGGTCGGAGTC-3′, R 5′-GAAGATGGTGATGGGATTTC-3′.

### 2.4 Mendelian randomization data sources

This study employed a two-sample MR approach followed by mediation analysis to investigate the potential causal effects of plasma protein levels on diabetic retinopathy and to explore intermediary biological mechanisms. Exposure data were obtained from the UK Biobank Pharma Proteomics Project (UKB-PPP), which profiled 2,923 plasma proteins in 54,219 participants and identified 14,287 primary genetic associations, 85% of which were novel (15). These high-confidence protein quantitative trait loci (pQTLs) served as genetic instruments for the MR analysis. Outcome data for diabetic retinopathy were derived from the FinnGen Release 12 GWAS, including 96,429 individuals (14,142 cases and 82,287 controls) of Finnish ancestry (16). To further explore potential mediators, we conducted a mediation analysis using immune cell data from the SardiNIA cohort (17), which includes 3,757 individuals with immune profiling.

### 2.5 Selection of instrumental variables

For MR analysis, IVs were selected based on three core assumptions: (1) they are strongly associated with the exposure (e.g., LAT2 expression); (2) they are not related to potential confounders; and (3) they affect the outcome (e.g., diabetic retinopathy) solely through the exposure (18, 19). For mediation MR, additional criteria were applied: the IV must influence the outcome only via the exposure and mediator, with no alternative pathways or unmeasured confounding between mediator and outcome (20, 21). SNPs were initially filtered using a genome-wide significance threshold of *p* < 5 × 10^−8^. In cases where fewer than 10 eligible SNPs were available, a relaxed threshold of *p* < 1 × 10^−5^ was adopted to ensure sufficient instrument strength. To ensure independence among SNPs, linkage disequilibrium (LD) pruning was performed using r² < 0.001 within a 10,000 kb window (22, 23). SNPs with an F-statistic < 10 were excluded to avoid weak instrument bias (24). All SNP harmonization, clumping, and F-statistic calculations were performed using the TwoSampleMR package in R (16).

### 2.6 Mendelian randomization analysis

We performed two-sample MR analysis to assess the causal effect of circulating protein levels on DR. The plasma pQTLs served as IVs. MR analyses were conducted using the R package TwoSampleMR (25, 26). For exposures with a single SNP, the Wald ratio method was applied, whereas for exposures with ≥2 SNPs, the inverse variance weighted (IVW) method was used. To account for multiple testing, *P* were adjusted using the false discovery rate (FDR) approach, and associations with FDR < 0.05 were considered significant.

### 2.7 Colocalization analysis

To validate whether the genetic signal for LAT2 protein levels shared a common causal variant with DR, we conducted colocalization analysis using cis-pQTL SNPs located within ±1 Mb of the LAT2 gene region (11, 27). Posterior probabilities were calculated for five hypotheses (H0–H4), where H4 represents a shared causal variant between protein expression and disease. Colocalization was considered significant when the posterior probability for H4 (PP.H4) exceeded 0.8. Analyses were performed using the coloc package in R, based on summary statistics from GWAS and proteomic datasets.

### 2.8 Phenome-wide Mendelian randomization analysis

To evaluate the potential broader effects of LAT2 expression across multiple phenotypes, we performed a phenome-wide MR (MR-PheWAS) analysis (28, 29). LAT2, identified as a candidate protective factor against diabetic retinopathy in the primary MR analysis, was selected as the exposure. The instrumental variables used for LAT2 were consistent with those applied in prior MR analyses. Summary-level outcome data were obtained from the Finngen R12 release (https://r12.finngen.fi/). The IVW or Wald ratio method was applied depending on the number of available SNPs. *P* values were corrected using the FDR method, and associations with Pfdr < 0.05 were considered statistically significant.

### 2.9 Statistical analysis

All MR analyses were conducted using the TwoSampleMR package in R (v4.3.3) (30). The IVW method was used as the primary analysis, and the Wald ratio was applied for single-SNP exposures (31–33). Sensitivity analyses included Cochran’s Q test for heterogeneity, MR-Egger intercept for pleiotropy, and leave-one-out analysis to assess the influence of individual SNPs (34, 35). In mediation analysis, a stepwise MR approach was used to evaluate the indirect effect of immune cells in the pathway from LAT2 to diabetic retinopathy. FDR correction was applied, and Pfdr < 0.05 was considered significant.

## 3 Results

### 3.1 Bidirectional Mendelian randomization reveals causal relationship between circulating proteins and diabetic retinopathy

**Figure 2:**
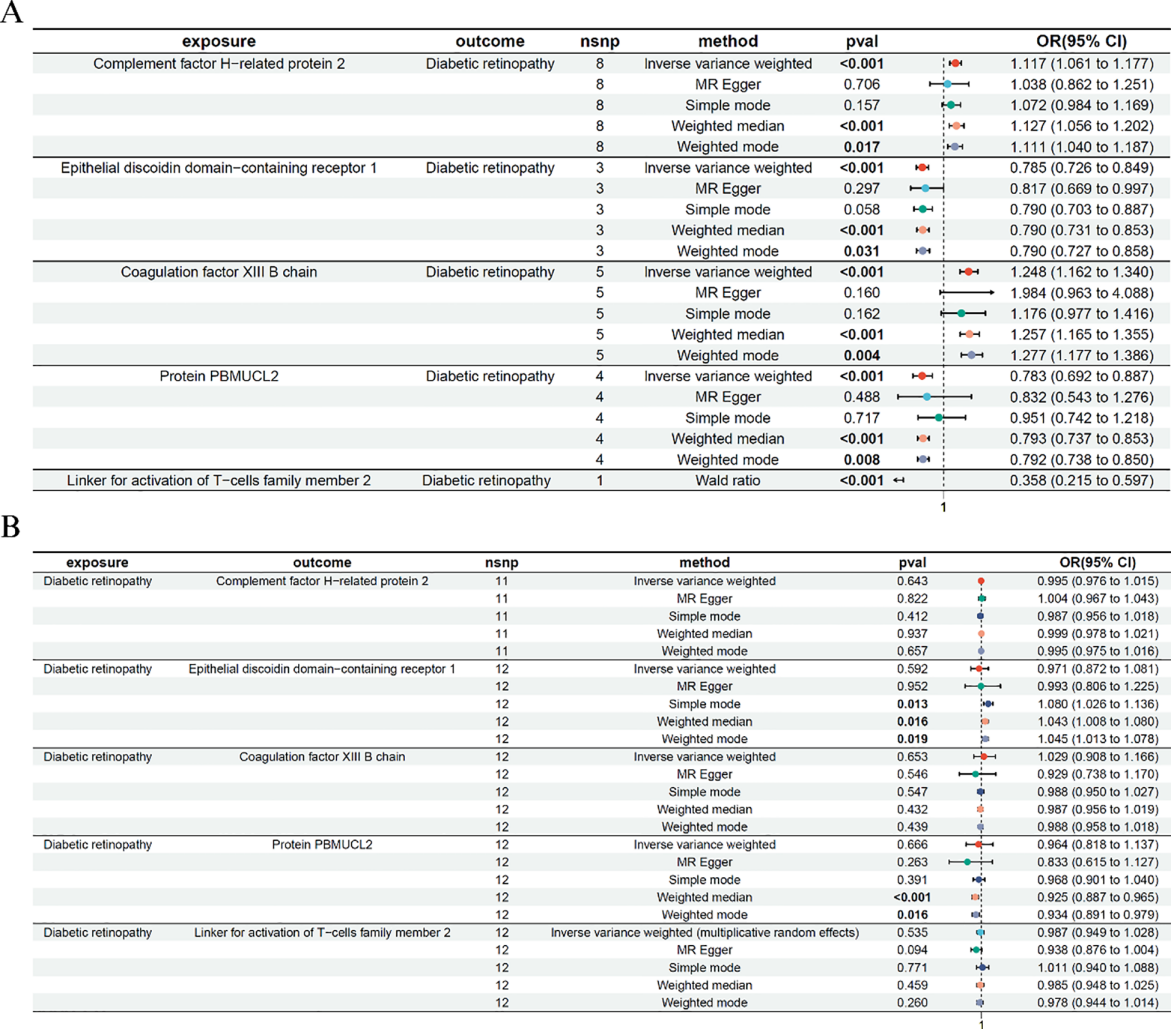
Bidirectional Mendelian randomization analysis between plasma proteins and diabetic retinopathy. **(A)** Forward MR analysis assessing the causal effects of plasma protein levels on diabetic retinopathy risk. The Wald ratio was used for LAT2 due to a single instrumental SNP. **(B)** Reverse MR analysis evaluating the effect of genetic liability to diabetic retinopathy on protein levels. Only significant IVW or Wald ratio results that passed heterogeneity and pleiotropy tests were retained. ORs and 95% CIs are shown.

We conducted bidirectional two-sample MR analyses to evaluate the causal relationship between plasma protein levels and DR. The forward MR (**Figure 2A**) examined the effects of genetically predicted protein levels on DR risk, while the reverse MR (**Figure 2B**) tested whether genetic liability to DR affected protein expression levels. The IVW method was used as the primary method and was tested for heterogeneity and horizontal polytropy (**Supplementary Tables S1**, **S2**).

In the forward MR analysis, five proteins—Coagulation factor XIII B chain, Epithelial discoidin domain-containing receptor 1, Complement factor H-related protein 2, Protein PBMUCL2, and the newly added Linker for activation of T-cells family member 2 (LAT2), showed significant causal associations with DR. For example, higher genetically predicted levels of Epithelial discoidin domain-containing receptor 1 were associated with a reduced risk of DR (IVW: OR = 0.785, 95% CI: 0.726–0.849, *p* < 0.001), while increased levels of Coagulation factor XIII B chain were associated with higher DR risk (IVW: OR = 1.248, 95% CI: 1.162–1.340, *p* < 0.001). Similar significant associations were observed for Complement factor H-related protein 2 and PBMUCL2. For LAT2, only one instrumental SNP passed the genome-wide significance threshold; thus, the Wald ratio method was used. The result showed that elevated LAT2 levels were significantly associated with reduced DR risk (OR = 0.358, 95% CI: 0.215–0.597, *p* < 0.001), suggesting a potential protective effect of this immune-related protein.

The reverse MR analysis revealed no significant effects of DR on protein levels, including LAT2, supporting a unidirectional association. This step served to validate the directionality of the observed effects.

### 3.2 Colocalization and transcriptome-based MR analyses identify LAT2 as a robust causal candidate

**Figure 3:**
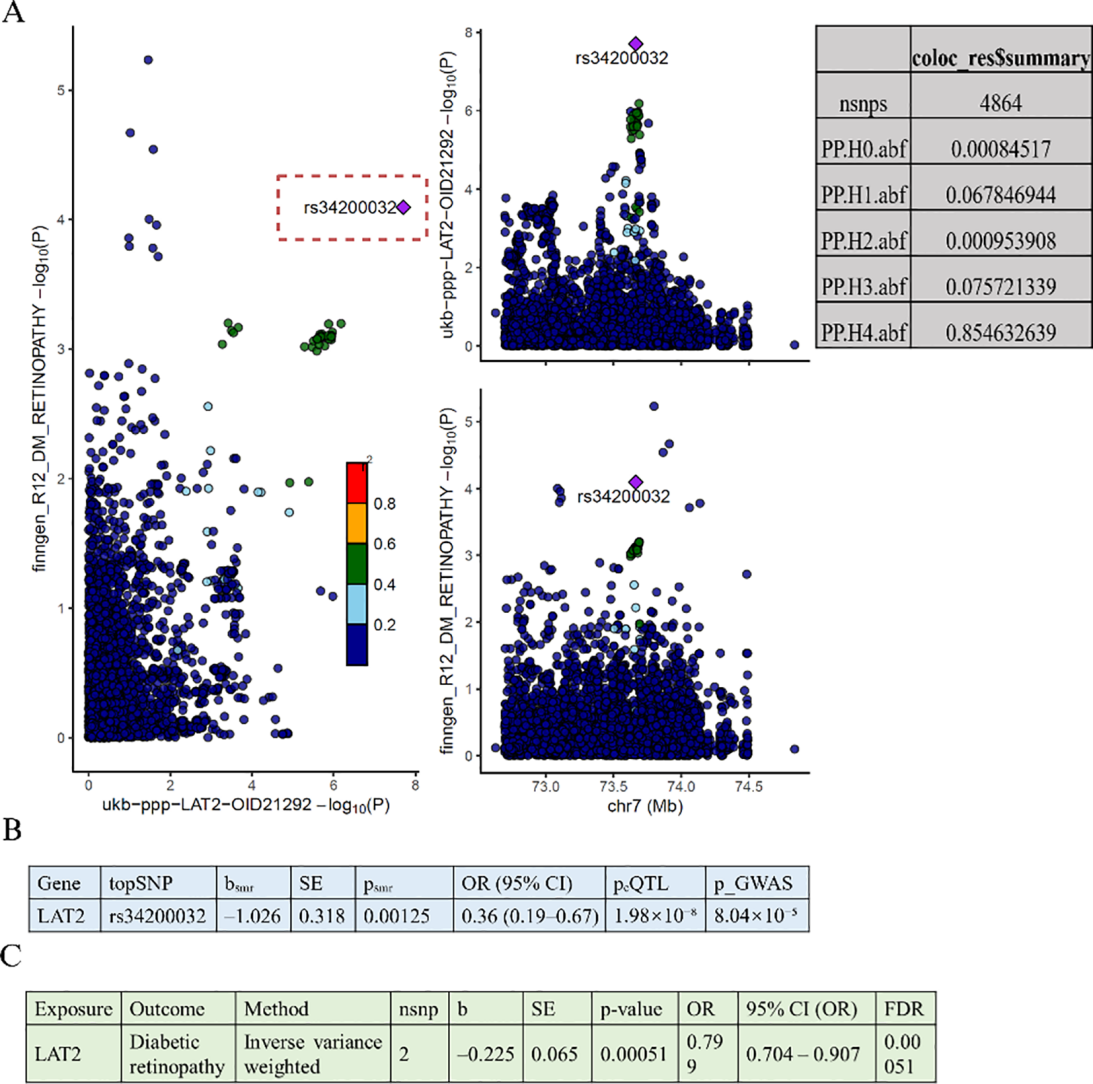
Colocalization and gene expression-based MR analyses of LAT2 and diabetic retinopathy. **(A)** Colocalization analysis shows a shared causal variant (rs34200032) between LAT2 protein levels and diabetic retinopathy, with high posterior probability (PP.H4 = 0.8546). **(B)** SMR analysis indicates that higher LAT2 expression is associated with lower risk of diabetic retinopathy (OR = 0.36, 95% CI: 0.19–0.67, p = 0.00125). **(C)** Two-sample MR using eQTLs confirms this association (OR = 0.80, 95% CI: 0.70–0.91, p = 0.00051, FDR = 0.00051).

Building upon the protein-level associations identified in **Figure 2**, we performed follow-up analyses to validate whether the observed MR signals could be supported by genetic colocalization and gene expression–based MR evidence. We systematically examined colocalization signals for the forward MR-identified proteins, but found that only LAT2 showed a high posterior probability of sharing a causal variant between protein levels and DR. Specifically, colocalization analysis for the LAT2 region (**Figure 3A**) revealed a strong posterior probability for hypothesis 4 (PP.H4 = 0.8546), suggesting a shared genetic signal. The lead SNP rs34200032 showed consistent effects in both the plasma proteomics and DR GWAS datasets.

To further assess whether genetically regulated expression of LAT2 contributes to DR risk, we conducted a SMR analysis (**Figure 3B**), which revealed a significant inverse association (b = –1.026, SE = 0.318, *p* = 0.00125), corresponding to an odds ratio of 0.36 (95% CI: 0.19–0.67). The lead SNP rs34200032 was significantly associated with LAT2 expression (eQTL *p* = 1.98 × 10^−8^) and DR risk (GWAS *p* = 8.04 × 10^−5^), further supporting its functional relevance.

To replicate this finding, we performed an eQTL-based two-sample MR using genome-wide significant variants (*p* < 5 × 10^−8^), a GWAS threshold of p < 0.001, and a 10,000 kb clumping window (**Figure 3C**). The results were consistent with the SMR analysis, again showing a protective effect of LAT2 expression on DR risk (IVW: *p* = 0.00051; OR = 0.799, 95% CI: 0.704–0.907).

### 3.3 Experimental validation of LAT2 in diabetic retinopathy models

To validate the findings from the MR and colocalization analyses, we next evaluated the expression and functional role of LAT2 in cellular models of DR. Based on previous results suggesting that LAT2 exerts a protective effect against DR, we hypothesized that its expression might be altered under hyperglycemic conditions.

**Figure 4:**
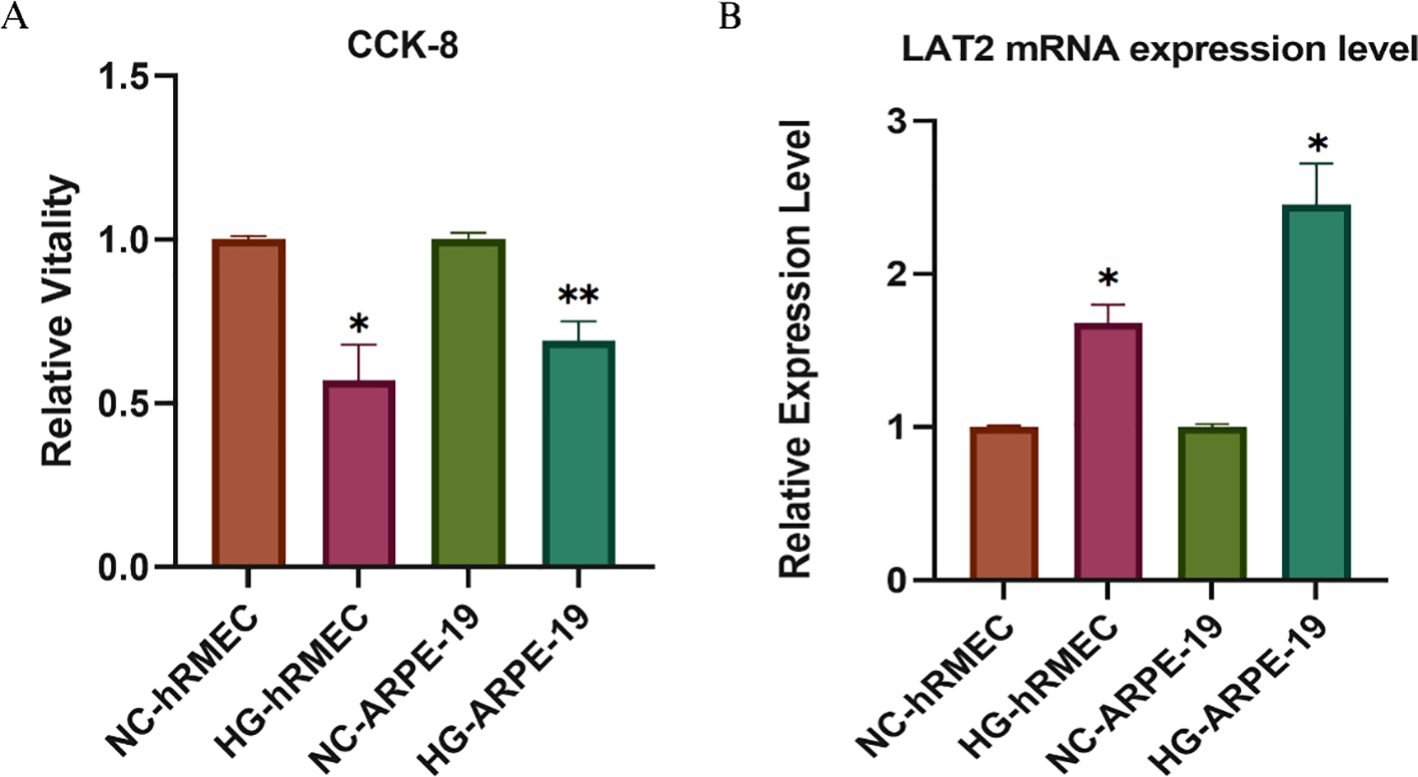
Validation of LAT2 expression and cell viability in retinal cells under high glucose conditions. **(A)** CCK-8 assay showing relative cell viability in hRMECs and ARPE-19 cells cultured under normal glucose (NC) or high glucose (HG, 48 h) conditions. **(B)** qPCR analysis of LAT2 mRNA expression. LAT2 expression was significantly upregulated in both hRMECs and ARPE-19 cells upon HG stimulation. Data are presented as mean ± SD (n = 3); *p < 0.05, **p < 0.01 vs. NC group.

As shown in **Figure 4A**, CCK-8 assays revealed that high glucose (HG) exposure significantly reduced cell viability in both hRMECs and ARPE-19 cells compared to their respective normal glucose (NC) controls (*p* < 0.05 or *p* < 0.01). This confirms the cytotoxic impact of hyperglycemia in retinal endothelial and epithelial cells. Interestingly, PCR analysis (**Figure 4B**) showed a significant upregulation of LAT2 mRNA levels under HG treatment in both hRMECs and ARPE-19 cells (*p* < 0.05), with a more pronounced elevation observed in ARPE-19. These results indicate a possible compensatory increase in LAT2 expression in response to glucose-induced stress, which aligns with the MR findings that suggest a protective effect of elevated LAT2 levels on DR risk.

### 3.4 Mediation Mendelian randomization reveals immune-mediated protective role of CD27^+^ memory B cells in LAT2–DR axis

Building on our findings that LAT2 is a protective factor against DR and upregulated under high-glucose conditions (**Figure 4**), we further explored its potential immunological mediators. Given the role of immune dysregulation in DR, we hypothesized that LAT2 might exert its protective effect partly via B-cell–related mechanisms.

**Figure 5:**
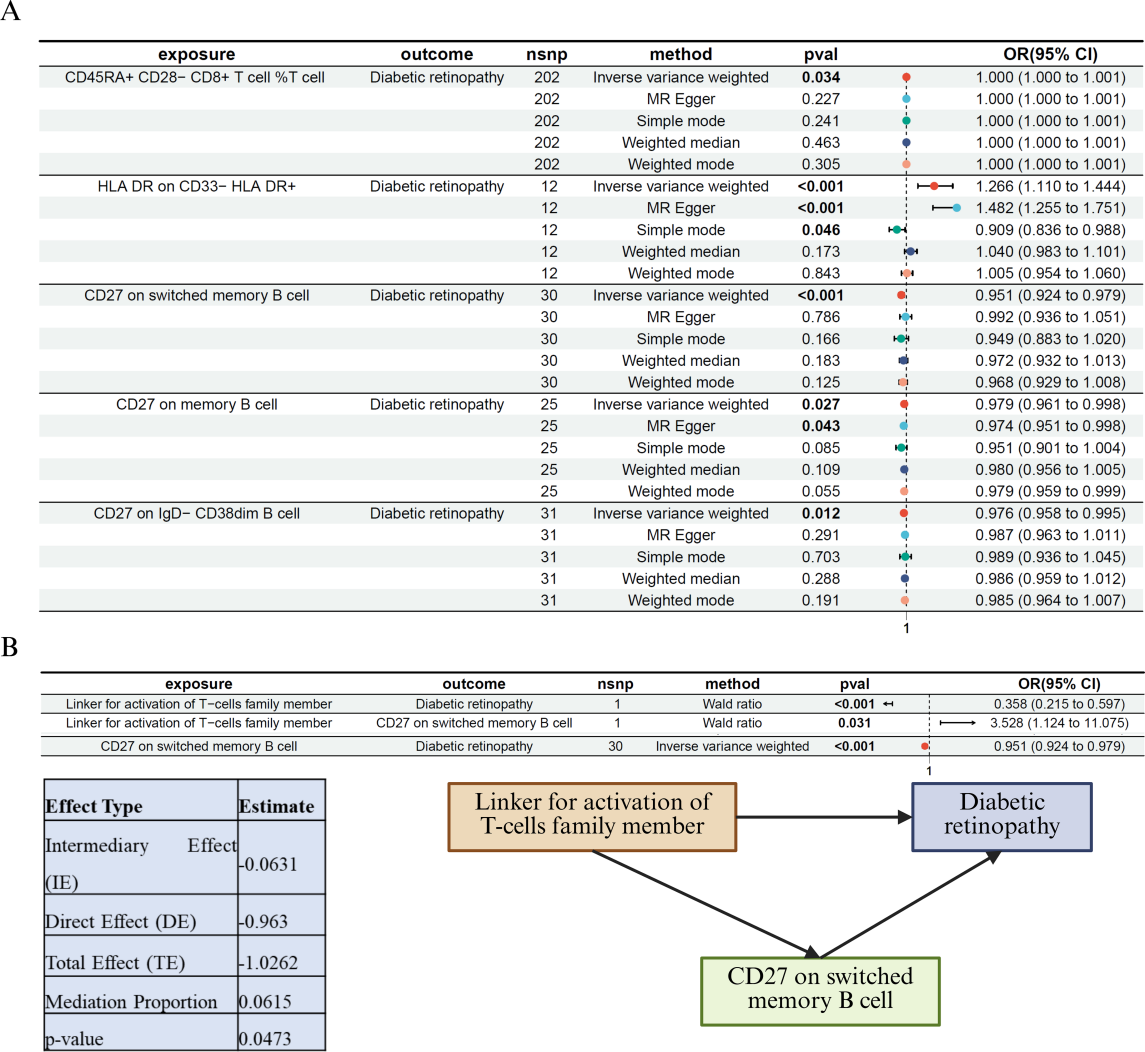
Immune cell–mediated Mendelian randomization and mediation analysis of LAT2 in diabetic retinopathy **(A)** MR analysis of immune cell traits and their associations with DR. Among the tested traits, CD27^+^ switched memory B cells were significantly and inversely associated with DR risk (*p* < 0.001), suggesting a protective effect. **(B)** Mediation MR analysis identifying CD27^+^ switched memory B cells as a partial mediator of the protective effect of LAT2 on DR. Left: a three-step MR framework depicting causal paths from LAT2 to DR via CD27^+^ B cells. Right: statistical summary of the indirect (mediated), direct, and total effects. The negative value of the indirect effect reflects the consistent protective direction of both LAT2 → B cell (positive association) and B cell → DR (negative association). The estimated mediation proportion was 6.2%, indicating a modest but statistically significant contribution of CD27^+^ B cells to the overall protective effect of LAT2.

To test this, we first performed MR analysis of immune cell traits on DR risk (**Figure 5A**). Among the tested traits, CD27^+^ switched memory B cells, CD27^+^ memory B cells, and CD27^+^ IgD^−^CD38^dim B cells showed nominally significant associations. Notably, higher proportions of CD27^+^ switched memory B cells were significantly associated with reduced DR risk (IVW: OR = 0.951, 95% CI: 0.924–0.979, *p* < 0.001).

We then applied a three-step MR-based mediation framework (**Figure 5B**). LAT2 was significantly associated with both DR risk (Wald ratio: OR = 0.358, 95% CI: 0.215–0.597, *p* < 0.001) and CD27^+^ switched memory B cell abundance (OR = 3.528, 95% CI: 1.124–11.075, *p* = 0.031). The B cell subset was, in turn, inversely associated with DR (OR = 0.951, 95% CI: 0.924–0.979, *p* < 0.001). The estimated indirect effect was –0.0631 (*p* = 0.0474), corresponding to a mediation proportion of 6.2%. The negative sign of the indirect effect reflects consistent directionality: increased LAT2 leads to increased protective B cells, which lowers DR risk. This supports the interpretation of a biologically meaningful partial mediation.

Together, these findings suggest that the protective effect of LAT2 against DR may be partly mediated by CD27^+^ switched memory B cells, highlighting the importance of adaptive immune pathways in the pathophysiology of diabetic retinopathy.

### 3.5 LAT2 exhibits tissue-specific association with diabetic retinopathy in a phenome-wide MR analysis

**Figure 6:**
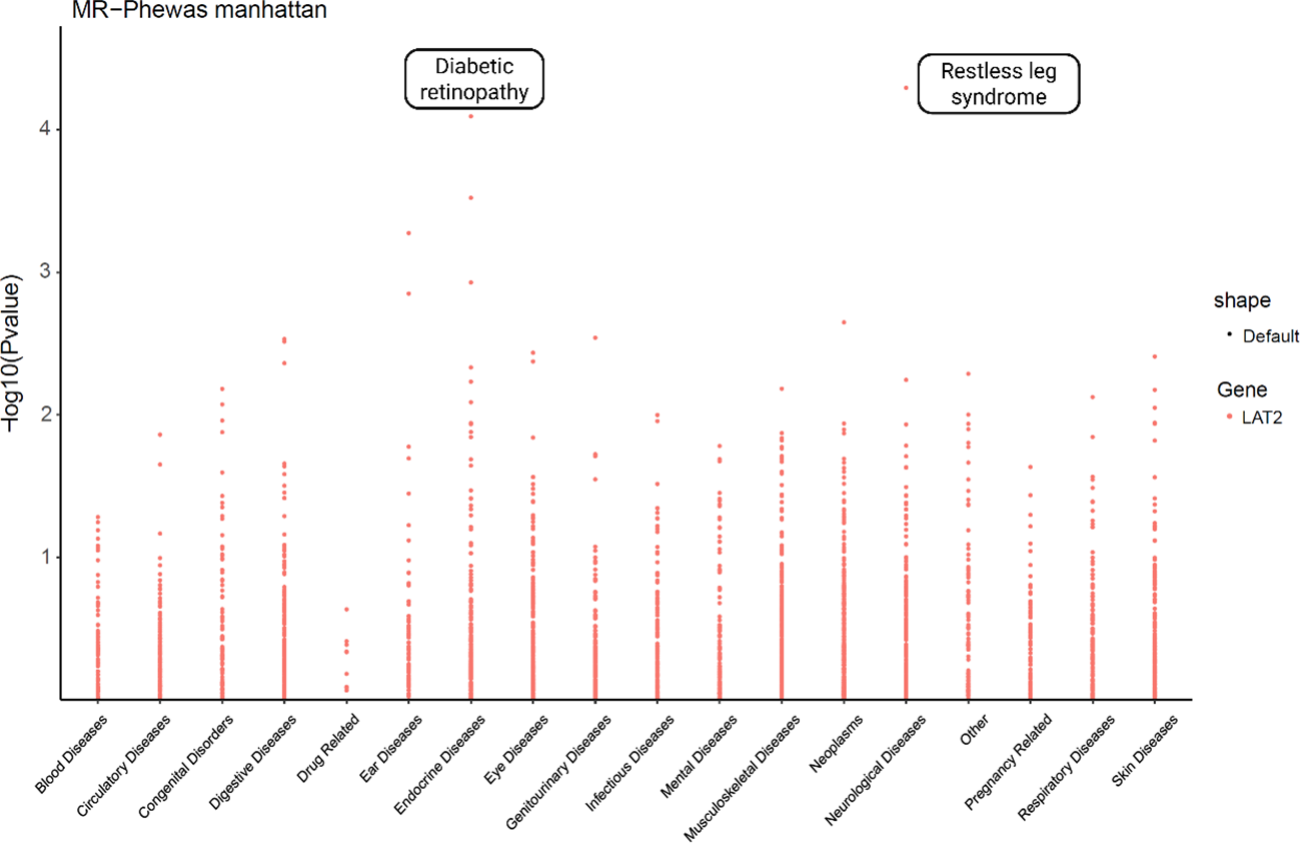
Phenome-wide Mendelian randomization analysis of LAT2 across disease categories. MR-PheWAS analysis was conducted to assess the potential causal associations between LAT2 and a broad range of phenotypes. The y-axis indicates the –log_10_-transformed p-values from MR tests, and phenotypes are grouped by disease categories on the x-axis. Notably, LAT2 showed the most significant association with diabetic retinopathy, supporting its specificity and functional relevance.

To further investigate the phenotypic specificity of LAT2’s protective effect, we performed a MR-PheWAS analysis across a broad range of disease traits. As shown in **Figure 6**, LAT2 expression exhibited significant associations with a limited number of phenotypes. Notably, diabetic retinopathy was among the top significant traits (*p* < 1×10^−4^), consistent with previous MR, colocalization, and eQTL-based results. In addition, we identified a potential association with restless leg syndrome, although its significance and biological plausibility require further investigation.

## 4 Discussion

In this study, we leveraged a two-sample MR framework integrated with colocalization, transcriptome-based MR, mediation analysis, and *in vitro* experiments to identify and validate causal plasma proteins involved in DR. Among the significant candidates, we highlight LAT2 as a protective factor against DR. This finding was supported by multiple layers of evidence, including genetic colocalization (PP.H4 = 0.8546), SMR and eQTL-based MR analyses, high-glucose cellular experiments, and MR-based immune mediation analysis.

Our results suggest that elevated circulating LAT2 levels confer a protective effect against DR, with an odds ratio of 0.358 in MR analysis. This effect was further substantiated at the transcriptional level, as genetically predicted LAT2 expression was inversely associated with DR risk. The colocalization analysis strongly supports a shared causal variant (rs34200032) between LAT2 expression and DR, reducing the possibility of confounding due to linkage disequilibrium or independent effects.

Functionally, LAT2 expression was significantly upregulated in human retinal endothelial and epithelial cells under hyperglycemic stress. This observation suggests a compensatory or protective upregulation of LAT2 in response to diabetic retinal injury, aligning with our MR findings. Given the known role of LAT2 in immune cell signaling and activation, it is plausible that LAT2 contributes to retinal homeostasis through immune modulation under diabetic conditions (36).

The mediation MR analysis further revealed that CD27^+^ switched memory B cells partially mediated the protective effects of LAT2 on DR, accounting for approximately 6.2% of the total effect. This finding implicates B-cell–related immune pathways in the protective mechanism of LAT2. While previous studies have emphasized the role of innate immune cells and pro-inflammatory cytokines in DR pathogenesis, our results extend this paradigm by highlighting the potential involvement of adaptive immune memory responses, particularly memory B cells (37, 38). These insights align with emerging evidence that immune cell dysfunction plays a crucial role in microvascular complications of diabetes (39–41).

Importantly, the phenome-wide MR analysis demonstrated the disease specificity of LAT2’s protective effect. Apart from DR, no other phenotypes exhibited significant associations after FDR correction, underscoring the tissue- and disease-specific relevance of LAT2 in the retinal vasculature and diabetic microangiopathy.

Despite these strengths, several limitations should be acknowledged. First, the use of European ancestry–based genetic datasets may limit the generalizability of our findings to other populations. Second, although our *in vitro* experiments support the upregulation of LAT2 under hyperglycemic stress, functional validation using LAT2 overexpression or knockdown models *in vivo* is warranted to elucidate its direct effects on retinal vascular integrity and immune regulation. Third, while five plasma proteins showed statistically significant causal associations with diabetic retinopathy in the forward MR analysis, only LAT2 passed all robustness checks, including Cochran’s Q test for heterogeneity. Some of the other proteins, such as Coagulation factor XIII B chain and Protein PBMUCL2, exhibited significant heterogeneity (Q-test *p* < 0.05), suggesting that their causal estimates may be affected by horizontal pleiotropy or variability in instrumental strength. These associations should therefore be interpreted with caution. Lastly, the mediation effect identified via CD27^+^ B cells accounted for a relatively small proportion of the total protective effect of LAT2, implying the involvement of additional cellular or molecular pathways that warrant further investigation.

In conclusion, our integrative MR study identifies LAT2 as a novel and specific protective plasma protein against diabetic retinopathy, likely acting through immune-mediated pathways involving memory B cells. These findings not only offer insights into the molecular etiology of DR but also present LAT2 as a promising candidate for biomarker development and future immunomodulatory interventions.

## Data Availability

The original contributions presented in the study are included in the article/**Supplementary Material**. Further inquiries can be directed to the corresponding author.
